# Teaching clinical reasoning by making thinking visible: an action research project with allied health clinical educators

**DOI:** 10.1186/1472-6920-14-20

**Published:** 2014-01-30

**Authors:** Clare Delany, Clinton Golding

**Affiliations:** 1 School of Health Sciences, The University of Melbourne, Melbourne, Australia; 2 Children’s Bioethics Centre, at the Royal Children’s Hospital, Melbourne, Australia; 3 Higher Education Development Centre, University of Otago, North Dunedin, New Zealand; 4 Honorary Senior Fellow of the Centre for Higher Education, University of Melbourne, Melbourne, Australia

**Keywords:** Clinical reasoning, Diagnostic reasoning, Clinical education, Professional development, Action research

## Abstract

**Background:**

Clinical reasoning is fundamental to all forms of professional health practice, however it is also difficult to teach and learn because it is complex, tacit, and effectively invisible for students. In this paper we present an approach for teaching clinical reasoning based on making expert thinking visible and accessible to students.

**Methods:**

Twenty-one experienced allied health clinical educators from three tertiary Australian hospitals attended up to seven action research discussion sessions, where they developed a tentative heuristic of their own clinical reasoning, trialled it with students, evaluated if it helped their students to reason clinically, and then refined it so the heuristic was targeted to developing each student’s reasoning skills. Data included participants’ written descriptions of the thinking routines they developed and trialed with their students and the transcribed action research discussion sessions. Content analysis was used to summarise this data and categorise themes about teaching and learning clinical reasoning.

**Results:**

Two overriding themes emerged from participants’ reports about using the ‘making thinking visible approach’. The first was a specific focus by participating educators on students’ understanding of the reasoning process and the second was heightened awareness of personal teaching styles and approaches to teaching clinical reasoning.

**Conclusions:**

We suggest that the making thinking visible approach has potential to assist educators to become more reflective about their clinical reasoning teaching and acts as a scaffold to assist them to articulate their own expert reasoning and for students to access and use.

## Background

Clinical reasoning is fundamental to all forms of healthcare practice [1], but it is difficult to teach because it is complex, situation specific, built up through experience and frequently based on tacit, automatic processes of pattern recognition [2-6]. It involves gathering and analyzing information (diagnostic reasoning) as well as deciding on therapeutic actions specific to a patient’s circumstances and wishes (therapeutic reasoning) [4]. It combines cognitive strategies such as analysis and problem solving with situated reasoning about patient needs in their broader clinical context [2,7]. Comparative studies of experts and novices have highlighted important differences in these thinking processes. Expert practitioners rely on experience to recognise patterns [3,6,8], and they tacitly and automatically integrate disciplinary knowledge, clinical data, and client preferences [3,4,7-9].

In contrast, novices do not have experts’ prior experience to enable them to automatically integrate information, so they work through a series of distinct and explicit thinking steps in a more fixed pattern [10]. They also tend to ask more questions than experts, some of which may be irrelevant to the situation or a particular patient’s care [9].

The inherent complexity of and experience required for expert clinical reasoning skills leads to two related challenges for teaching clinical reasoning. First, clinical educators who are experienced clinicians may find it difficult to explain and teach clinical reasoning because it has become ingrained in their way of thinking and being for them [3,11-13]. Second, students may find it hard to grasp because it is effectively invisible and inaccessible to them [14,15]. In this article we introduce and evaluate (via a pilot action research project with allied health educators), an approach to teaching clinical reasoning based on the pedagogical method of making thinking visible [16,17]. The goal, when applied to teaching clinical reasoning is to assist educators to use a type of metacognition (thinking about their own thinking) [18] to reveal the otherwise ‘hidden’ elements of their reasoning [19], as an explicit scaffold to guide their students’ thinking and reasoning.

Standard approaches to teaching clinical reasoning have focused on broad thinking steps including to ‘gather information from a range of different sources’; ‘state the likely diagnosis’; ‘describe the presenting pattern of symptoms’ and ‘decide the most appropriate management for a particular patient’ [1,5,7,20]. However, because these broad steps provide little concrete detail about what types of knowledge a clinician is drawing from and how she or he is interpreting and synthesizing that knowledge, [14]. They may still be too abstract and detailed and some of the nuanced thinking steps of clinical reasoning may remain invisible or at least inaccessible for the learner.

The teaching method of ‘making thinking visible’ has previously been used successfully in classroom settings to promote and guide student thinking [21]. It involves identifying and then ‘repackaging’ the thinking steps used by experts when they engage in clinical reasoning into ‘thinking routines’. Thinking routines consist of short, repeatable actions that isolate a type of thinking and provide heuristics or ‘tools’ for enabling and promoting this thinking [21]. For example, a commonly used routine to encourage evaluative thinking is ‘Plus, Minus and Interesting’ or PMI [22]. When using this routine, students first consider the plus or positives, then the minus or negatives, and finally, any interesting questions or issues that arise. By doing these three actions students engage in evaluative thinking that is both expansive and inclusive. By repeating these actions regularly and frequently – making it a routine – they become more skilful at evaluation, until it becomes an automated way of thinking for them [17].

To make the structure of their thinking visible, clinical educators first identify the types of knowledge they are privileging, the cognitive processes they are using and the connections they are making in their mind. They then refine this thinking to concrete steps or thinking routines which capture the specific clinical context. (See Table 1 for detailed steps of this approach, as well as Golding [17] for the underlying principles). This approach is similar to other strategies that have been shown to be effective for teaching clinical reasoning, such as ‘think out loud’ [4] and using concept maps [23,24].

**Table 1:** Making thinking visible

**Principle**	**Action**
1. Articulate	• Make explicit the thinking required
	• Reverse engineer your own thinking. Explain and describe how you think through problems and issues
2. Make concrete and visible	• Identify thinking behaviours – what expert thinkers ask and say when they engage in thinking
4. Refine, chunk & sequence	• Refine and group the thinking behaviours into useful heuristics – thinking routines
5. Enculturate	• Make the thinking a routine part of your teaching
	• Repeat and model thinking routines
	• Encourage students to frequently and regularly use these routines

Three key pedagogical principles underpin this approach. The first recognises that reducing complex expert thinking to a thinking routine that a student can use, is a form of simplification of knowledge to reduce the cognitive work of clinical reasoning [25-27]. Simplification of knowledge is not designed to ignore or reduce the inherent complexity of clinical reasoning, but rather to provide an entry point for students to participate in disciplinary thinking and discourse. A second pedagogical premise is that students can be effectively facilitated to learn by participating in the daily activities of their community of practitioners, where peers, role models and mentors scaffold or extend learning through guidance, modelling and discussion [28,29]. Lave and Wenger [30] describe this conception of learning as a type of professional socialisation and Vygotsky’s [31] theories about the importance of explicit scaffolding and social inclusion of students is also educationally relevant [32-34]. The third pedagogical premise is that when educators think about their own thinking, they are engaging in reflective and metacognitive thinking Schön [35], and this assists them to develop a more explicit understanding of their own clinical reasoning prior to teaching others [36], even if their clinical reasoning is partly subconscious [11].

## Methods

We used action research – a method widely used in primary and secondary education as a powerful means of professional learning for clinical educators [37]. The key tenets of action research are that real problems are discussed with the intention of improvement and empowerment [38]. Action research engages participants in a structured process of reflection [35] about their teaching, so they can generate new knowledge about their teaching practices [39]. This is consistent with the idea of mindfulness in education, described by Ritchhart and Perkins [16] as having an open and creative state of consciousness, in contrast to a passive, inert and superficial learning disposition. We purposefully chose action research methodology to enable participants to actively reflect on their styles and methods of clinical supervision and teaching [40]. Standard methods of professional learning for clinical teaching include lectures and workshops about teaching strategies [41-44]. While there is evidence that educators benefit from participating in lectures and short workshops [27,41,45], concerns remain that such courses may not provide sufficient opportunity for teachers learn through active participation.

Because action research empowers participants to construct, use and evaluate their own knowledge and understanding [46,47], it provides both a practical and theoretical frame for clinical educators to link their clinical thinking expertise to their teaching methods. Using an iterative learning cycle [48,49], it encourages them to construct their own teaching practices by developing, trialling and evaluating new teaching methods.

**Figure 1:**
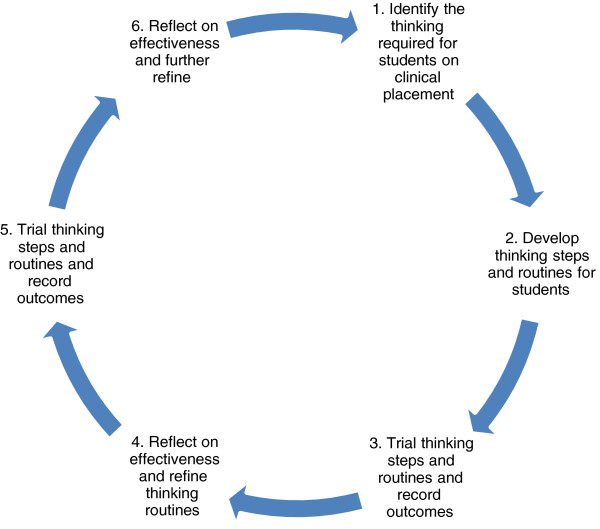
The cycle of action research.

In our study (Figure 1), clinical educators reflected on how to teach clinical reasoning, using the lens of making thinking visible to focus their reflection. They were then encouraged to evaluate and refine how they teach clinical teaching within their own practice with the two authors and with their peers in the discussion sessions.

The participants were recruited in the following way: We introduced the ‘Making thinking visible’ teaching approach (Table 1) at three 2-hour seminars at a large Australian metropolitan teaching hospital in May 2010. We surveyed the 70 allied health professionals who attended about how they teach clinical reasoning, and this is reported elsewhere [50]. In the third seminar, we invited the audience to indicate interest in participating in action research/learning project by leaving their contact details in a box at the back of the lecture theatre.

Twenty-one clinical educators from eight allied health disciplines (physiotherapy, social work, podiatry, occupational therapy, education play therapy, music therapy, prosthetics, and speech pathology), with an average of ten years clinical practice experience, and eight years clinical supervision provided their contact details. The participants all worked in one of three large public hospitals in Melbourne, Australia. All participants were involved in supervising undergraduate students, except for those from social work, where supervision involved postgraduate students. They attended an average of three sessions of a possible 7 (see Table 2). Ethics approval was obtained from The University of Melbourne Human Research Ethics Committee.

**Table 2:** Action research participants – discipline and numbers

**Participant discipline**	**Participant numbers**						
	**Session 1**	**Session 2**	**Session 3**	**Session 4**	**Session 5**	**Session 6**	**Session 7**
Education play therapy	1	2					
Music therapy		1	1			1	
Occupational therapy	2	2	1	2			
Physiotherapy	9	9	7	3	4	4	4
Podiatry	1	1					
Prosthetics	1	1	1		1		1
Social work		1	1	1	1		
Speech pathology	1	1		1			
**Total**	**15**	**18**	**11**	**7**	**6**	**5**	**5**

The action research followed a structured pattern of participant reflection and trialing of teaching heuristics (Figure 1):

### Stage one and two

Participants were asked to identify an area of clinical reasoning or practice their students found challenging, or an area where their students rarely engaged in the necessary reasoning. They were then asked to identify how they would go about clinical reasoning in this same situation by stating the steps they followed when they consciously think through the same challenges faced by their students. The final step involved refining the steps into a ‘thinking routine’ that students could employ. We facilitated this process of reflection by asking the clinical educators to consider and discuss the following questions:

•What questions would you ask yourself if you faced a challenging clinical scenario?

•What type of thinking would you like your students to develop?

•What would your students ask if they were engaged in this thinking?

•What questions can you ask to encourage this thinking?

•How can you turn these questions and thinking steps into a thinking routine/heuristic – a short, repeatable set of questions or actions that isolates and engages the same type of thinking?

The thinking routines that emerged typically comprised three short questions or single words, which gave concrete actions or thinking steps for students to follow (Table 3). These were listed on a whiteboard and checked with participants.

**Table 3:** Examples of developing and refining thinking routines

**Clinical activity**	**Initial thinking routine**	**Evaluation of this thinking routine**	**Refined thinking routine**
1. Assessing a patient with a musculoskeletal injury (Physiotherapy)	1. *Gather* information	“This routine was too complex for novices… students still struggled with what to *consider*”	*Consider:*
	2. *Consider* diagnosis		*1. Underlying* structures
	3. *Sort* priorities		*2. Connecting* structures
			*3. Patterns* of pain and symptoms
2. Reassessment of a child after initial treatment (Physiotherapy)	1. *List* main problems	“This routine was too challenging for a student who could only *list* but could not make the connections or make sense of how the previous treatment impacted on the patient.”	1. *What changes* occurred in the child’s symptoms since last presentation?
	2. *Compare* with previous problems		2. *What is the impact* of treatment on their mobility, function, muscle tone… ?
	3. *Decide* whether to continue with same treatment		3. *Whether* to continue or change your treatment?
3. Assessing a limb for prosthesis fitting (Prosthetics and orthotics)	1. *Feel* stump	“This routine really helped students. I further refined this with sub questions”	1. *Feel* what is the tissue consistency? What are the anatomical prominences?
	2. *Describe* shape, texture		2. *Describe* what is the profile of the residuum? Is it bulbous?
	3. *Test* lining		3. *What* is the distal circumference?
			4. *Test* does the skin pull in against the gel when rolled on?
4. Treatment planning (Social work)	1. *Why* are you/they here?	“This routine was still too complex for some students. Asking ‘what’ about a patients’ goals also involves sorting and categorising the goals”	No new routine but…
	2. *What* are this patient’s goals?		“The routine meant we discussed the importance of asking open-ended questions to clients and then using their answers to build further questions”
	3. *Summarise* what you could do		

### Stage three

Participating educators then trialed these routines with their students. They were asked to record the thinking routines they trialled; to state their teaching goal for using the routine; and to describe in concrete terms what happened after using the routine. They were also asked to evaluate the impact of using the thinking routine on student responses and on their own teaching styles by responding to the following questions:

•What went well?

•What didn’t go so well?

•What did the students say or do?

•Was their behaviour different? If so how?

•Did the student engage in clinical reasoning? How was this demonstrated?

•Have the routines impacted on your clinical education practice? If so how?

### Stage four

Educators’ written reflections were compared in the discussion sessions and they evaluated and further refined their thinking routine to better match their students’ learning needs. Any changes to thinking routines were recorded on the whiteboard and checked with participants.

### Stage five

The educators then trialed the refined routines.

### Stage six

Participating educators evaluated and further refined these routines.

Stage three and five of the action research cycle occurred during the participants’ clinical teaching. Stage one, two, four and six occurred through individual reflection, regular email interaction with the researchers, and during fortnightly discussion sessions. All participants engaged in the individual reflection. Five to eighteen participants attended each discussion session where they worked in multidisciplinary groups of three to four, and each participant attended an average of three sessions (Table 2). Participants took as many sessions as needed until they were satisfied that the routines they had refined would foster the relevant clinical thinking in their students.

### Data analysis

Two sources of data were obtained and analysed. First, educators’ written reflections and observations of the thinking routines they developed and trialled, what happened during and after using the routine and the impact of the action research on their clinical teaching. Second, the researchers’ memos during the discussion groups documenting different types of thinking routines including refinements made over time (Table 3).

We used content analysis to summarise educators’ descriptions of their teaching trials and our own discussion group memos [51-53]. Both authors summarized and grouped the educators’ written reports into categories about teaching clinical reasoning and then refined these categories to reach a consensus of two overriding descriptive themes about the impact of using the ‘making thinking visible approach’ their clinical teaching. The first was an orientation by participating educators towards students’ understanding of the reasoning process. The second was heightened awareness of personal teaching styles and approaches to teaching clinical reasoning.

## Results

### Theme 1: a focus on student understanding – through developing and refining thinking routines

Table 3 provides examples of thinking routines documented by participating educators and recorded by the researchers in the discussion groups. These examples demonstrate how participants refined their thinking routines when the initial formulation did not work for the student. Sometimes they judged that the routine did not fit the clinical situation or did not adequately support their students’ thinking and so it was abandoned in favour of a different routine.

“The routine didn’t really fit the scenario … On reflection I would use a slightly different routine myself with an inpatient such as that.” (music therapist)

In other cases educators noticed that a particular step of a routine was too difficult for a student and so they refined this step, as illustrated in the first example in Table 3. In yet other cases, the entire routine was refined because it did not encourage the thinking needed for a particular aspect of a clinical task (see Table 3).

### Theme 1: a focus on student understanding – noticing how routines influenced student reasoning

The participating educators documented examples of how they used the thinking routines to engage students in specific strategies of clinical reasoning and how students responded:

“The three words allowed for more concise documentation and kept her on track. It was useful in refining and reducing complex issues.” (social worker)

“Both of the students were able to reflect on the thinking routine and explain how it had helped to guide their assessment of the infant.” (physiotherapist)

“The student was able to go into more detail when asking questions.” (social worker)

Some of the more common observations were that after using the thinking routines, students began to justify their clinical judgements, explain their reasoning, and attempt to distinguish between clinical presentations:

“After they had tried to prioritise their problems, the students justified their choices to me by explaining the reasoning behind them.” (physiotherapist)

“Using the routine, meant the student was able to reduce the complex issues that the patient presented with and focus on the fundamental issues at hand specifically for the patient.” (social worker)

“The student did engage in clinical reasoning because they wanted to know what is normal, that is, to have a point of comparison to know the significance of their assessment findings.” (physiotherapist)

The educators also reported that the routines acted as a prompt, and provided a structure or framework for students’ thinking:

“The student reported that the routine ‘gave structure in my head’, and that it helped with on the spot thinking, especially using the prompt ‘clarify’.” (physiotherapist)

“The student appreciated a structure to work with and was encouraged by having a strategy since she had struggled with other placements.” (occupational therapist)

“The student was able to use the word cues from the routine to identify what they saw and heard during the session, it prompted them to tease out the specific details.” (podiatrist)

According to educators’ reports, some students became more focussed, confident and independent in their thinking, and had better management of their time as a result of using the thinking routines:

“Normally when you say to students: ‘please try to prioritise your problem list and then show it to me’, they immediately want to ask me the answers and talk it through with me. However, with these two students, they very quietly went about trying to prioritise themselves, using the thinking routine and without asking me at all.” (physiotherapist)

“The student showed initiative with making plans and was more assertive with patients. Having a stronger plan allowed her to focus more on other elements of her interaction with patients.” (speech pathologist)

### Theme 2: awareness of teaching styles for clinical reasoning

A focus on student thinking seemed to encourage the participating educators to become more specific in articulating the thinking steps they wanted their students to develop. They became more discerning about what they expected their students to learn, which they encapsulated in their thinking routines. They also developed greater awareness of the current thinking and reasoning of their students. They identified concrete instances of student thinking when it occurred:

“I noticed that she first wrote out a list of problems and then changed the order to reflect the correct prioritised order. I could really see her thinking through the process.” (physiotherapist)

“The student watched and picked up on ‘non-verbal’ cues the patient was giving from the ‘look’ prompt. She was able to describe in detail what went on in the session, both verbally and non-verbally.” (social worker)

“The word ‘describe’ worked really well in asking the student to be really detailed about what they had seen and heard. This included being specific about the movements the client did, how they did them and how they explained this.” (physiotherapist)

The participating educators also reported noticing when students had missed an important aspect of clinical reasoning, and they reflected on how they could encourage this missing thinking:

“The student needed extra prompts to pick out the key issues.” (educational play therapist)

“She is struggling to ascertain what the patient’s problems are after her assessment, let alone prioritise them. So I think she wasn’t ready for this thinking routine, it is too advanced. She needs a thinking routine to help her work out the patient’s problems. This thinking routine I introduced to her today would be ideal (I think!) for a student who can get the main problems, but who is struggling to prioritise them.” (physiotherapist)

### Theme 2: refining teaching styles for clinical reasoning

Participating educators recognised specific teaching opportunities and were motivated to continue with trialling and refining their teaching:

“I am more willing to try new things as it did work – the challenge is finding the right ‘set’ or ‘routine’ for the particular student.” (music therapist)

“Using this particular thinking routine has encouraged me to think about how I can devise other thinking routines for the other clinical areas I work in.” (educational play therapist)

“Once the undergraduate students return I would like to be able to try this thinking routine with them. I think it would be better to use it in a clinic that is less busy to allow more time and opportunity for the student to feedback on the process, and to gauge the usefulness of this thinking routine for them.” (physiotherapist)

## Discussion

In this action research project we trialled a method for teaching clinical reasoning. Participating educators developed a tentative heuristic of their own clinical reasoning, trialled it with students, evaluated if it helped their students to reason clinically, and then refined it so the heuristic was targeted to developing each student’s reasoning skills. As predicted by action research theories [50,54-56], the cycle of - developing, trialling, evaluating and refining – resulted in participating educators taking responsibility for their own specific professional learning [46]. It also encouraged them to examine the impact of their teaching on student learning [35,49,57-61]. This outcome reflects a key tenet of learning derived from action research. Concrete experience is the impetus for creating knowledge through a process of “observing and reflecting on that experience, forming abstract concepts and generalisations, and testing the implications of these new concepts in new situations” ([62], p.46).

Although aspects of expert clinical reasoning are considered to be subconscious and impossible to precisely describe [11], the clinical educators in our study were able to make visible and accessible some steps in their reasoning process by reflecting on what they would do or say in a specific clinical situation [3]. The multidisciplinary nature of discussion groups assisted in this process, because in order to describe their clinical thinking to a colleague from a different discipline, educators had to be more concrete and explicit about their knowledge and reasoning. The focus of the clinical educators shifted during their involvement in the project, from considering a) What their students should know and do; to encompass b) What students currently understood and did; to also include reflection on a third level c) How to enable students to move from their current understandings and behaviours to the desired learning outcomes. Recognising and moving through these essential elements of teaching [21,36], is an example of what Shulman refers to as developing pedagogical content-knowledge [63]. They became involved in actively constructing their own discipline’s curriculum to build student understanding of clinical reasoning [61].

The specific focus on creating thinking routines seemed to be useful for teaching clinical reasoning because it directed the clinicians to develop an understanding of their own clinical reasoning, which is a necessary precondition for *teaching* clinical reasoning [17,64]. It provided a method of working towards the pedagogical goal of aligning the learning outcome of developing expert clinical reasoning skills with specific teaching methods - in this case - the thinking steps used by expert clinicians [65,66]. Clinical educators were also able to use the routines to prompt their students to engage in independent clinical reasoning, rather than have them passively watch and wait for the answers [65,67].

These results suggest that making expert clinical thinking visible is a potentially valuable approach for assisting to bridge the gap between expert and novice reasoning [10,14,68]. The routines encouraged educators to provide students with access to their specific disciplinary language and to assist them to become part of their profession’s community of clinical practice [28-32]. They accord with the successful use of this method in classroom settings [21]. However, unlike the classroom research where routines were given to teachers, the thinking routines developed in this research were derived through action research from clinician/teachers’ own ‘expert’ thinking and were specifically focused on facilitating steps of thinking for disciplinary-specific clinical reasoning.

There are several important limitations to this research project. The overall sample of participating educators is limited to eight allied health disciplines with small numbers of participants. There was both a variable and declining attendance at each discussion session, which participating educators explained as being caused by changing student supervision loads and busy clinical commitments. Also, seven discussion groups of one-hour duration, scheduled every two weeks is a relatively small amount of time to effect a sustained change in teaching behaviour. A further factor which limits both generalizability and replication of this study is the dynamic and responsive nature of discussions between the authors, as facilitators, and the participants in each focus group session. The impact on students’ actual clinical reasoning capacities was not measured because the data comprised of educators’ descriptions of students’ responses and reasoning. Data which relies on participants’ descriptions and interpretations of their teaching practice, are always open to differing and subjective interpretations and reports [69,70].

Despite these limitations, we suggest that the key pedagogical tenets of the making thinking visible’ approach are potentially useful for clinical educators to assist in teaching students the steps of clinical reasoning. The making thinking visible approach encourages educators to become more reflective about their clinical reasoning teaching and acts as a scaffold to assist them to articulate their own expert reasoning and for students to access and use. The approach requires further testing and evaluating for its impact on clinical reasoning performance and in specific disciplines and clinical settings.

## Conclusions

How can clinical educators learn to teach clinical reasoning, given it is second-nature to them, but inaccessible and unobservable to students? Our conclusion is that the making visible thinking approach in combination with an action research methodology could be useful as a form of professional learning. It guides educators to be learning and improvement oriented, to be explicit about their own clinical reasoning, and to develop and trial strategies to support student reasoning.

## Competing interests

The authors declare that they have no competing interests.

## Authors’ contributions

CD and CG contributed equally to all aspects of the research and writing. Both authors read and approved the final manuscript.

## Authors’ information

Clare Delany (PhD) is Associate Professor and Director of Teaching and Learning at the School of Health Sciences, The University of Melbourne and Senior Ethics Associate at the Children’s Bioethics Centre, at the Royal Children’s Hospital. Research interests include clinical education pedagogy and practice and clinical ethics consultation and education.

Clinton Golding (PhD) is Senior Lecturer at the Higher Education Development Centre, University of Otago, an Honorary Senior Fellow of the Centre for the Study of Higher Education, University of Melbourne, and the Chair of the Higher Education Research and Development Society of Australasia, New Zealand. Research interests include educating for thinking across the disciplines, especially clinical reasoning in the medical disciplines and health professions.

## Pre-publication history

The pre-publication history for this paper can be accessed here:

http://www.biomedcentral.com/1472-6920/14/20/prepub

## References

[B1] Norman G Research in clinical reasoning Med Educ 2005 39 4 418 427 10.1111/j.1365-2929.2005.02127.x 15813765

[B2] Ajjawi R Higgs J Core components of communication of clinical reasoning Advances in Health Sci Educ 2012 17 1 107 119 10.1007/s10459-011-9302-7 21638086

[B3] Eva KW What every teacher needs to know about clinical reasoning Med Educ 2005 39 1 98 106 10.1111/j.1365-2929.2004.01972.x 15612906

[B4] Durning SJ Artino AR Jr Pangaro LN van der Vleuten C Schuwirth L Context and clinical reasoning Advances in Health Sci Educ 2011 45 9 927 938 10.1111/j.1365-2923.2011.04053.x 21848721

[B5] Ryan S Higgs J Higgs J, Jones M, Loftus S, Christensen N Teaching And Learning Clinical Reasoning Clinical Reasoning In The Health Professions 2008 3 Amsterdam: Elsevier 379 87

[B6] Ark TK Brooks LR Eva KW The benefits of flexibility Med Educ 2007 41 3 281 287 10.1111/j.1365-2929.2007.02688.x 17316213

[B7] Higgs J Jones M Loftus S Christensen N Clinical Reasoning In The Health Professions 2008 3 Amsterdam: Elsevier

[B8] Edwards I Jones M Carr J Braunack-Mayer A Jensen G Clinical reasoning strategies in physical therapy Phys Ther 2004 84 4 312 330 15049726

[B9] Higgs J Burn A Jones M Integrating clinical reasoning and evidence-based practice Am Assoc of Critical-Care Nurses 2001 12 4 482 490 10.1097/00044067-200111000-00005 11759421

[B10] Charlin B Tardif J Boshuizen HP Scripts and medical diagnostic knowledge: theory and applications for clinical reasoning instruction and research Academic Med 2000 75 2 182 190 10.1097/00001888-200002000-00020 10693854

[B11] Bargh J Unconscious thought theory and its discontents Soc Cogn 2011 29 6 629 647 10.1521/soco.2011.29.6.629

[B12] Reilly BM Inconvenient truths about effective clinical teaching Lancet 2007 370 9588 705 711 10.1016/S0140-6736(07)61347-6 17720022

[B13] Hill S Troublesome knowledge: why don’t they understand? Health Inf Libr J 2010 27 1 80 83 10.1111/j.1471-1842.2010.00880.x 20402808

[B14] McAllister L Rose M Higgs J, Jones M, Loftus S, Christensen N Speech-Language Pathology Students: Learning Clinical Reasoning Clinical Reasoning In The Health Professions 2008 Amsterdam: Elsevier 397 404

[B15] Fish D de Cossart L Thinking outside the (tick) Box: rescuing professionalish and professional judgment Med Educ 2006 40 403 404 10.1111/j.1365-2929.2006.02441.x 16635118

[B16] Ritchhart R Perkins DN Life in the mindful classroom: nurturing the disposition of mindfulness J Soc Issues 2000 56 1 27 47 10.1111/0022-4537.00150

[B17] Golding C Educating for critical thinking Higher Educ Res and Develop 2011 30 3 357 379 10.1080/07294360.2010.499144

[B18] Epstein A Shulman L Sprafka S Medical Problem Solving: An Analysis Of Clinical Reasoning 1978 Cambridge, MA: Harvard University Press

[B19] Lempp H Seale C The hidden curriculum in undergraduate medical education British Med Jour 2004 329 7469 770 773 10.1136/bmj.329.7469.770 PMC520997 15459051

[B20] Gay S Bartlett M McKinley R Teaching clinical reasoning to medical students Clin Teach 2013 10 308 312 10.1111/tct.12043 24015736

[B21] Ritchhart R Church M Morrison K Making Thinking Visible 2011 San-Fransisco: Jossey Bass

[B22] de Bono E Serious Creativity 1992 New York: HarperBusiness

[B23] Novak JD Canas AJ Theoretical origins of concept maps, how to construct them and uses in education Reflecting Education 2007 3 1 29 42

[B24] Daley BJ Torre DM Concept maps in medical education: an analytical literature review Med Educ 2010 44 5 440 448 10.1111/j.1365-2923.2010.03628.x 20374475

[B25] La Rochelle JS Durning SJ Pangaro LN Artino AR van der Vleuten CP Schuwirth L Authenticity of instruction and student performance Med Educ 2011 45 8 807 817 10.1111/j.1365-2923.2011.03994.x 21752077

[B26] Van Merriënboer J Sweller J Cognitive load theory and complex learning Educ Psych Rev 2005 17 2 147 177 10.1007/s10648-005-3951-0

[B27] Dhaliwal G Developing teachers of clinical reasoning Clin Teach 2013 10 313 317 10.1111/tct.12082 24015737

[B28] Mamede S Schmidt H The structure of reflective practice in medicine Med Educ 2004 38 1302 1308 10.1111/j.1365-2929.2004.01917.x 15566542

[B29] Ajjawi R Higgs J Learning to reason: a journey of professional socialisation Advances in Health Sci Educ 2008 13 2 133 150 10.1007/s10459-006-9032-4 17288004

[B30] Lave J Wenger E Situated learning: Legitimate Peripheral Participation 1991 Cambridge: Cambridge University Press

[B31] Vygotsky L Mind In Society: The Development Of The Higher Psychological Processes 1978 Cambridge: Harvard University Press

[B32] Clouder L Becoming professional: exploring the complexities of professional socialization in health and social care Learn Health Soc Care 2003 2 4 213 222 10.1046/j.1473-6861.2003.00052.x

[B33] Egan T Jaye C Communities of clinical practice Health 2009 13 1 107 125 19103718 10.1177/1363459308097363

[B34] Wenger E Communities Of Practice 1998 Cambridge: Cambridge University Press

[B35] Schön D The Reflective Practitioner 1983 New York: Basic books

[B36] Kell C Jones L Mapping placement educators’ conceptions of teaching Physiotherapy 2007 93 4 273 282 10.1016/j.physio.2006.11.011

[B37] Cohen L Manion L Morrison K Morisson KRB Research Methods In Education 2007 Chicago: Psychology Press

[B38] Dewar B Sharp C Using evidence: how action learning can support individual and organisational learning through action research Educ Action Res 2006 14 2 219 237 10.1080/09650790600718092

[B39] McNiff J Whitehead J All You Need To Know About Action Research 2006 London: Sage

[B40] Burchell H Dyson J Action research in higher education: exploring ways of creating and holding the space for reflection Educ Action Res 2005 13 2 291 300 10.1080/09650790500200280

[B41] Notzer N Abramovitz R Can brief workshops improve clinical instruction? Med Educ 2008 42 152 156 10.1111/j.1365-2923.2007.02947.x 18309569

[B42] Kilminster S Jolly B Effective supervision in clinical practice settings Med Educ 2000 34 827 840 10.1046/j.1365-2923.2000.00758.x 11012933

[B43] van de Ridder J Stokking K McCaghie W ten Cate O What is feedback in clinical education? Med Educ 2008 42 189 197 10.1111/j.1365-2923.2007.02973.x 18230092

[B44] Yeates PJA Stewart J Barton JR What can we expect of clinical teachers? Med Educ 2008 42 2 134 142 10.1111/j.1365-2923.2007.02986.x 18230087

[B45] Weurlander M Stenfors-Hayes T Developing medical teachers’ thinking and practice Higher Educ Res and Develop 2008 27 2 143 153 10.1080/07294360701805283

[B46] Crow J Smith L Keenan I Journeying between the Education and Hospital Zones in a collaborative action research project Ed Action Research 2006 14 2 287 306 10.1080/09650790600718258

[B47] Trevitt C Learning in academia is more than academic learning: action research in academic practice for and with medical academics Educ Action Res 2008 16 4 495 515 10.1080/09650790802445676

[B48] Harland T University Teaching 2012 London: Routledge

[B49] Kolb D Experiential Learning 1984 USA: Prentice-Hall

[B50] Delany C Golding C Bialocerkowski A Teaching for thinking in clinical education Focus on Health Prof Educ 2013 14 2 44 56

[B51] Graneheim UH Lundman B Qualitative content analysis in nursing research: concepts, procedures and measures to achieve trustworthiness Nurse Educ Today 2004 24 2 105 112 10.1016/j.nedt.2003.10.001 14769454

[B52] Downe-Wamboldt B Content analysis: method, applications, and issues Health Care Women Int 1992 13 313 321 10.1080/07399339209516006 1399871

[B53] Liamputtong P Qualitative data analysis Health Promot J Austr 2009 20 2 133 139 19642962 10.1071/he09133

[B54] Zuber-Skerritt O Professional Development In Higher Education 1992 London: Kogan Page Ltd

[B55] McNiff J Action Research: Principles And Practice 1988 London: Routledge

[B56] Carr W Kemmis S Becoming Critical 1986 London: The Falmer Press

[B57] Cohen D Nisan M, Schremer O Professions Of Human Improvement: Predicaments Of Teaching Educational Deliberations 2005 Jerusalem: Keter 278 294

[B58] Swanwick T See one, do one, then what? faculty development in postgraduate medical education Postgrad Med J 2008 84 993 339 10.1136/pgmj.2008.068288 18716011

[B59] Delany C Watkin D A study of critical reflection in health professional education Advances in Health Sci Educ 2009 14 3 411 429 10.1007/s10459-008-9128-0 18528774

[B60] Ho A Watkins D Kelly M The conceptual change approach to improving teaching and learning Higher Educ 2001 42 2 143 169 10.1023/A:1017546216800

[B61] Bleakley A Curriculum as conversation Advances in Health Sci Educ 2009 14 3 297 301 10.1007/s10459-009-9170-6 19521789

[B62] Zuber-Skerritt O Improving learning and teaching through action learning and action research High Ed Res and Develop 1993 12 1 45 58 10.1080/0729436930120105

[B63] Shulman L Knowledge and teaching: foundations of the new reform Harv Educ Rev 1987 57 1 1 22

[B64] Atkinson K Ajjawi R Cooing N Promoting clinical reasoning in general practice trainees: role of the clinical teacher Clin Teach 2011 8 176 180 10.1111/j.1743-498X.2011.00447.x 21851565

[B65] Delany C Bragge P A study of physiotherapy students’ and clinical educators’ perceptions of learning and teaching Med Teach 2009 31 9 402 411 10.1080/01421590902832970 19811176

[B66] Biggs J Enhancing teaching through constructive alignment Higher Ed 1996 32 3 347 364 10.1007/BF00138871

[B67] Hafler JP Ownby AR Thompson BM Fasser CE Grigsby K Haidet P Kahn MJ Hafferty FW Decoding the learning environment of medical education Academic Med 2011 86 4 440 10.1097/ACM.0b013e31820df8e2 21346498

[B68] Jensen GM Gwyer J Shepard KF Hack LM Expert practice in physical therapy Phys Ther 2000 80 28 43 10623958

[B69] Green J Britten N Qualitative research and evidence based medicine BMJ 1998 316 7139 1230 1232 10.1136/bmj.316.7139.1230 9583929 PMC1112988

[B70] Morse J Singleton J Exploring the technical aspects of “Fit” in qualitative research Qualitative Health Res 2001 11 6 841 847 10.1177/104973201129119424 11710081

